# Climbing-inspired twining electrodes using shape memory for peripheral nerve stimulation and recording

**DOI:** 10.1126/sciadv.aaw1066

**Published:** 2019-04-19

**Authors:** Yingchao Zhang, Ning Zheng, Yu Cao, Fengle Wang, Peng Wang, Yinji Ma, Bingwei Lu, Guohui Hou, Zizheng Fang, Ziwei Liang, Mengkun Yue, Yan Li, Ying Chen, Ji Fu, Jian Wu, Tao Xie, Xue Feng

**Affiliations:** 1 AML, Department of Engineering Mechanics, Tsinghua University, Beijing 100084, China.; 2 Center for Flexible Electronics Technology, Tsinghua University, Beijing 100084, China.; 3 State Key Laboratory of Chemical Engineering, College of Chemical and Biological Engineering, Zhejiang University, Hangzhou 310027, China.; 4 Institute of Flexible Electronics Technology of THU, Jiaxing 314000, China.

## Abstract

Peripheral neuromodulation has been widely used throughout clinical practices and basic neuroscience research. However, the mechanical and geometrical mismatches at current electrode-nerve interfaces and complicated surgical implantation often induce irreversible neural damage, such as axonal degradation. Here, compatible with traditional 2D planar processing, we propose a 3D twining electrode by integrating stretchable mesh serpentine wires onto a flexible shape memory substrate, which has permanent shape reconfigurability (from 2D to 3D), distinct elastic modulus controllability (from ~100 MPa to ~300 kPa), and shape memory recoverability at body temperature. Similar to the climbing process of twining plants, the temporarily flattened 2D stiff twining electrode can naturally self-climb onto nerves driven by 37°C normal saline and form 3D flexible neural interfaces with minimal constraint on the deforming nerves. In vivo animal experiments, including right vagus nerve stimulation for reducing the heart rate and action potential recording of the sciatic nerve, demonstrate the potential clinical utility.

## Teaser

Proposed 3D neural electrode can self-climb onto peripheral nerve driven by body temperature and form flexible neural interface.

## INTRODUCTION

**Fig. 1:**
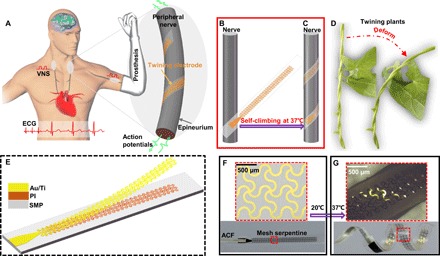
Twining electrodes for PNS. (**A**) Schematic diagram of the conceptual PNS neuromodulation for restoring the motor and physiological functions (left) and the electrode-nerve interface (right). (**B** and **C**) Concept of the self-climbing process from the flattened state driven by body temperature. (**D**) Photographs of the twining plants during deformation. (**E**) Layout of the proposed twining electrode. (**F**) Twining electrode in the temporarily flattened state. ACF, anisotropic conductive film. (**G**) Twining electrode that recovered from its temporary shape (inner diameter of ~2 mm). Photo credit: Yingchao Zhang, Tsinghua University.

The peripheral nervous system (PNS) plays a crucial role in communication between the central nervous system and various motor and sensory end-plates (Fig. 1A). Hence, new therapeutic strategies for treating various refractory diseases through electrode-nerve interfaces have been developed, such as motor nerve stimulation and recording for controlling prosthetics and vagus nerve stimulation (VNS) for treating partial epilepsy, depression, heart failure (HF), and hypertension (*1*, *2*).

To decrease damage to nerves and increase long-term stability, several forms of extraneural electrodes have been proposed to replace intraneural electrodes, which penetrate into the nerve fascicles (*3*, *4*). Although promising, existing extraneural electrodes have at least one of the following drawbacks: large mechanical and geometrical mismatches and complicated surgical implantation procedures. Limited by traditional two-dimensional (2D) planar processing, the first-generation extraneural electrodes, including traditional 3D cuff electrodes, helical electrodes, and flat interface nerve electrodes, are made of silicone rubber (thickness, ~0.5 mm; modulus, ~2 MPa) and platinum (thickness, ~25 μm; modulus, ~200 GPa) (*5*–*8*). Large mechanical and geometrical mismatches still exist at the neural interfaces. If the traditional 3D cuff/helical electrodes are sufficiently small, then the requirement for effective contact with the nerves will be satisfied. However, the electrode may cause serious compression on the nerves, especially for child patients whose peripheral nerves are immature and will further grow after the electrode implantation. Although making the inner diameter of the cuff/helical electrodes larger than that of the nerves can partially release the compression, it may induce more damage due to the friction on the neural interfaces during movement. Moreover, poor contact also leads to higher interfacial impedance; thus, stimulation efficiency and the recorded signal-to-noise ratio (SNR) will be reduced. Reducing the thickness of the substrate and the metal layer can decrease the mechanical mismatch to some extent, such as the more recently developed 2D extraneural electrodes, which are based on polyimide (PI) and parylene-C (*9*–*12*). However, compared to the neural tissues (modulus, ~100 kPa) (*13*), all of these extraneural electrodes are still not soft enough (modulus, ~2 GPa) and require complicated surgical fixation. Because of the hard-to-change geometries, both traditional silicone rubber–based 3D cuff/helical electrodes with predefined stiff structures and PI-based electrodes with 2D planar structures have difficulties integrating with 3D peripheral nerves following surgical implantation. These drawbacks may induce irreversible damage to the nerves and cause serious biological issues, including inflammation, demyelination, axonal degradation, and blood vein compression (*14*–*17*). Several adverse reactions caused by these drawbacks have been reported clinically, such as vomiting, cough, and difficulty in breathing in VNS therapy (*18*, *19*).

Twining plants, which are commonplace in nature, first use their flexible apical stem to find support by circumnutational movements and then adaptively form a spiral configuration depending on the size of the support to maintain stability (*20*). In this way, flexible twiners can grow upward to capture more sunshine and other resources, without losing stability even under large deformation (Fig. 1D and movie S1). This symbiotic phenomenon of natural selection has attracted much research from different disciplines since Darwin’s era (*20*–*22*) and gives us considerable inspiration to solve the above problems. Here, inspired by these twining plants, we developed a biocompatible twining electrode, with the capability of self-climbing driven by body temperature and of forming conformal neural interfaces. On the basis of traditional 2D planar processing and transfer printing technology, the twining electrodes are fabricated by integrating stretchable mesh serpentine wires onto a flexible shape memory substrate (Fig. 1E) and reconfigured to a 3D helix to match the 3D peripheral nerves. Before surgical implantation, the twining electrodes are temporarily flattened to a 2D planar state. Driven by 37°C normal saline (NS), the temporarily flattened twining electrodes can naturally self-climb onto the nerves, forming 3D flexible neural interfaces (Fig. 1, B and C). Furthermore, no additional surgical fixation is required. During the deformations of the peripheral nerves such as swelling, bending, and stretching, the highly flexible and stretchable twining electrodes can naturally deform with the nerves without damage.

The key materials used in our approach are intelligent shape memory polymers (SMPs) capable of permanent shape reconfigurability, distinct elastic modulus controllability, and shape memory recoverability driven by body temperature. The design principle of the SMP network is illustrated in fig. S1. With the introduction of dynamic covalent bonds into the polymer network, the permanent shape of traditional thermoset SMPs can be reconfigured (*23*, *24*). This advanced development offers a new strategy for designing 3D twining electrodes that are compatible with the traditional 2D planar processing but without being limited by molds. Figure 1 (F and G) shows the two states of the twining electrode, i.e., the flattened state and the twined state, respectively. By contrast, most of the existing electronics based on SMPs are not very compatible with traditional 2D planar processing, which limits the development of high-precision electronics (*23*, *25*–*28*). Distinct elastic modulus controllability from ~100 MPa to ~300 kPa before and after the phase transition greatly facilitates the surgical implantation in limited space, where the twining electrodes are handled in a relatively stiff state and soften after climbing on the nerves. No surrounding tissues are burned during the shape memory process because we have designed the transition temperature (*T*_trans_) of the SMPs to be ~37°C, close to human body temperature. We show precursors for the synthesis of the SMPs in fig. S2, and we describe the synthesis in detail in the Materials and Methods.

## RESULTS

### Fabrication process

**Fig. 2:**
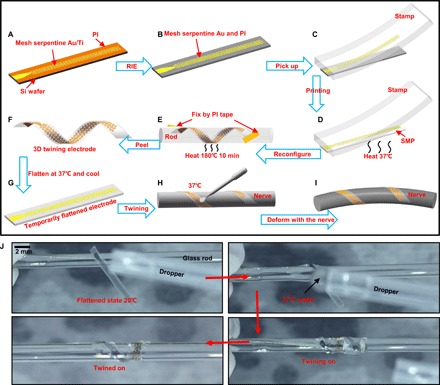
Schematic illustrations of the detailed fabrication process of the twining electrode and images of the self-climbing process. (**A** and **B**) Mesh serpentine design of the Au/Ti layer and the PI film, respectively. (**C** and **D**) Transfer printing process of the mesh serpentine Au/Ti/PI from Si onto the SMP substrate. (**E** and **F**) Reconfiguration of the permanent shape from the 2D planar shape to the designed 3D helical shape. (**G** to **I**) Schematic illustrations of the surgical implantation processes of the twining electrode with the aid of the shape memory effect. (**J**) Images of the in vitro experiments and the self-climbing processes of the twining electrode on a glass rod. Photo credit: Yingchao Zhang, Tsinghua University.

Figure 2 schematically demonstrates the concept and the associated fabrication paradigm. The fabrication process of the twining electrode is divisible into three main parts: fabrication of the mesh serpentine (Fig. 2, A and B), transfer printing process (Fig. 2, C and D), and 3D helical structure formation process (Fig. 2, D to F). Fabrication of the mesh serpentine starts with spin casting of an ultrathin PI film (~2 μm) (fig. S3) onto silicon wafers coated with a layer of a sacrificial polymethylmethacrylate (PMMA) film. After that, layers of 10-nm Ti and 200-nm Au (fig. S3) are deposited on the surface of the cured PI film by electron beam evaporation, followed by traditional lithography and etching to design the mesh serpentine structure (Fig. 2A). To further decrease the bending and tension stiffness of the whole structure, we also etched the PI film into the same mesh serpentine pattern by reactive ion etching (RIE), as shown in Fig. 2B. Then, by dissolving the sacrificial layer (PMMA) with acetone, the meshed Au/Ti/PI is picked up quickly with an elastic stamp (Fig. 2C), printed slowly onto the SMPs in a 2D planar state (*29*), and connected with the anisotropic conductive film. To facilitate the printing process, the SMPs are heated above the *T*_trans_ but below the plasticity temperature (*T*_p_) to enhance the surface adhesion (Fig. 2D) (*30*). After the transfer printing process, the initial 2D planar assembly is twined on a rod with a radius of *r*_0_, and the two ends are fixed by PI tape (Fig. 2E). The designed radius *r*_0_ is approximately equal to the radius of the peripheral nerves for the conformal contact. High-temperature (180°C) heating for 10 min (fig. S4D) leads to the reconfiguration of the permanent shape to the desired 3D helical shape (Fig. 2F). During this high-temperature procedure, the thermoplastic PI film is also reshaped into a helical structure, which will promote the recovery of the twining electrode, as proven in note S1. To facilitate the subsequent integration with the nerve, we flattened the twining electrode at 37°C and then cooled it down to obtain the temporarily flattened electrode (Fig. 2G). Driven by 37°C NS, the temporarily flattened electrode can thus self-climb onto the nerve to recover its permanent helical configuration and conformally make contact with the nerve (Fig. 2, H and I). The network transformations of the SMPs corresponding to the fabrication process (Fig. 2, D to H) are illustrated in fig. S2. The sequence of images in Fig. 2J and movie S2 demonstrates the self-climbing processes of the twining electrode in an in vitro experiment. We performed the electrical conductivity tests for the fabricated twining electrodes with inner radii of ~0.5 mm (~126 ohms) and ~1 mm (~119 ohms), as shown in movie S3.

### Material characterization and structure optimization

**Fig. 3:**
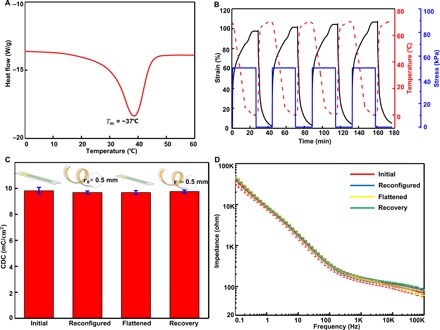
Materials characterization. (**A**) DSC curve for the synthetic SMPs. (**B**) Consecutive elasticity (shape memory) cycles. (**C** and **D**) CDC and impedance spectroscopy of the electrode under four different states.

Unlike traditional extraneural electrodes (*2*, *31*) and most flexible electronics (*32*–*37*), the materials chosen for the insulating substrates of the developed twining electrodes are a class of smart materials, i.e., the SMPs. The differential scanning calorimeter (DSC) curve for the synthetic SMPs (Fig. 3A) shows that the melt transition temperature (*T*_m_) is approximately 37°C (i.e., *T*_trans_ = ~37°C), which means that the SMPs recovering their permanent shapes can be actuated by body temperature (movie S4). This advantage not only vastly simplifies the surgical implantation process but also greatly reduces the risk of tissue burns during shape recovery. The consecutive shape memory cycles in Fig. 3B were obtained under stress-controlled mode with identical deformation. Both the shape fixity and shape recovery ratios are above 97% within four cycles, which are good enough for the twining electrode because only one shape recovery cycle is required in actual use (Fig. 2, F to H). Other advantages, such as large fracture strain (~1100%), low initial elastic modulus (~300 kPa), fine thermal stability, and favorable reconfigurability (fig. S4), make the developed SMPs highly suitable as the smart substrates of the twining electrodes. In addition, the biocompatibility of the main precursors in our SMPs has been previously demonstrated (*38*, *39*). Characterization of the electrochemical interface is also conducted to evaluate the twining electrodes by two vital parameters, i.e., charge delivery capacity (CDC) and impedance. CDC and impedance spectroscopy of electrodes under four different states, i.e., initial flat state, reconfigured state, flattened state, and recovery state, were obtained from cyclic voltammogram (CV) and electrochemical impedance spectroscopy (EIS), respectively, as shown in Fig. 3 (C and D). The results show that both the CDC and impedance values change little before and after the reconfiguration and twining processes (i.e., corresponding to Fig. 2, D and F to H) (see figs. S5 and S6 for more details) because only a small strain (~0.24%) appeared in the Au layer during the deformations, as we calculated below. In addition, the average CDC (~9.7 mC/cm^2^) and the impedance magnitude (~156 ohms) at 1 kHz are comparable to those of previously reported extraneural electrodes (*12*, *40*). The twining electrode can be used for both recording and stimulation. A lower impedance magnitude is more suitable for both electrical recording and stimulation, i.e., increasing the recorded SNR and the stimulation efficiency. A larger CDC can decrease the current amplitudes that are required to activate the nerves during the stimulation, which can also decrease the electrical damage to the nerve (*10*, *40*, *41*). Therefore, much effort has been devoted to decreasing the impedance and increasing the CDC by enlarging the surface area, such as by roughening the surface, or by adopting new materials such as iridium oxide (IrO_*x*_), carbon nanotubes, and poly(3,4-ethylene-dioxythiophene) (PEDOT) (*41*, *42*). It should be pointed out that the reported materials with high surface areas are compatible with the developed fabrication process as long as they can withstand the high *T*_p_.

**Fig. 4:**
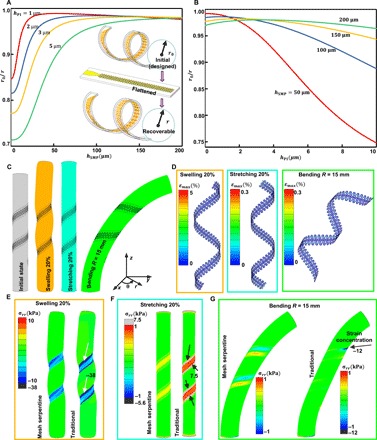
Structure optimization. (**A**) *r*_0_/*r* versus *h*_SMP_ at several different *h*_PI_. (**B**) *r*_0_/*r* versus *h*_PI_ at several different *h*_SMP_. (**C**) FEA models of three deformations of the nerve. (**D**) Maximum strain in the Au layer under the three deformations. (**E** to **G**) Comparisons of the normal stress applied on the nerve between the traditional helical electrode and the twining electrode under the three deformations.

Structural optimization aims to improve recoverability, minimize the maximum strain in the Au layer $\varepsilon_{\text{Au}}^{\text{max}}$, and decrease the constraints on the nerves. Recoverability is defined as the ratio of the initial (designed) radius *r*_0_ to the final recoverable radius *r* (the inset in Fig. 4A). The specific expressions have been derived in note S1. The calculated results show that, for a given thickness of PI *h*_PI_, *r*_0_/*r* first increases and then decreases with increasing SMP thickness *h*_SMP_; for a given *h*_SMP_, *r*_0_/*r* first increases and then decreases with increasing *h*_PI_ as well (Fig. 4, A and B, and fig. S7B). A higher *r*_0_/*r* leads to a more convenient surgical implantation procedure, less dependence on interfacial adhesion, and improved conformability (see note S1 for more details). Figure S7C gives $\varepsilon_{\text{Au}}^{\text{max}}$ versus *h*_SMP_ and *h*_PI_ under a bending radius of 0.5 mm. A smaller $\varepsilon_{\text{Au}}^{\text{max}}$ means that less fracture or yield of the Au layer will occur during the fabrication and twining processes (Fig. 2, E and G). For a comprehensive consideration of the above two aspects and the feasibility of the fabrication process, the selected *h*_SMP_ and *h*_PI_ are ~2 and ~100 μm, respectively, which are practical in terms of both clinical applications and fabrications. Here, *r*_0_/*r* is 0.98, and $\varepsilon_{\text{Au}}^{\text{max}}$ is 0.24%. With decreasing *h*_SMP_ and *h*_PI_, bending stiffness *EI* and stretching stiffness *EA* diminish markedly because *EI* and *EA* are proportional to the third and first power of the thickness, respectively. The *EI* values of traditional silicone rubber– and PI-based extraneural electrodes are usually ~2.1 × 10^−7^ N·m^2^ and ~4.6 × 10^−10^ N·m^2^, respectively (*5*, *8*, *9*, *43*), while the *EI* value of the twining electrodes is ~1.0 × 10^−10^ N·m^2^ (note S2). By taking advantage of the mesh serpentine design (*44*–*46*), the *EA* value is greatly decreased to 0.08 N (fig. S8); comparatively, the *EA* values of traditional silicone rubber– and PI-based extraneural electrodes are ~5000 and 40 N, respectively (note S3). With this structural optimization design, less constraint will be applied on the nerves during the growth and deformation process. We confirm this by finite element analysis (FEA) (fig. S9). Figure 4C describes the three deformations of the nerve, where a 20% swelling deformation is used to imitate the nerve growth process of child patients, and 20% stretching and bending (*R* = 15 mm) deformations are used to imitate the deformations of the nerves during movement. The maximum strain $\varepsilon_{\text{Au}}^{\text{max}}$ is far less than the fracture strain of Au (5%) during the swelling process and is less than the yield strain of Au (0.3%) during the stretching and bending deformations (Fig. 4D) (see Materials and Methods for details). Movie S5 qualitatively demonstrates the mechanical reliability of the twining electrode under large stretching and bending deformations. The FEA results (Fig. 4, E to G, and fig. S9) show that, compared with the traditional cuff electrodes, helical electrodes, and non-meshed electrodes, the twining electrodes minimize the mechanical mismatch and apply much lower stresses (including the normal pressure and the shear stress) on the nerves during all three of the abovementioned deformations. In addition, the applied stresses on the nerve and the reliability of the Au layer can be further improved by increasing the stretchability of the twining electrode according to previous works (*44*–*47*).

### Vagus nerve stimulation

**Fig. 5:**
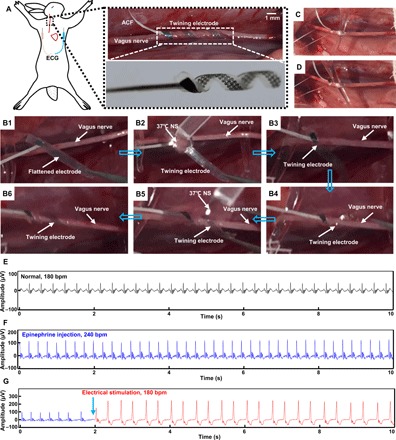
Photographs and ECG data from the in vivo VNS animal experiments. (**A**) Schematic diagram of VNS and recording of ECG (left) and images of an implanted twining electrode (inner diameter of 1 mm) on the vagus nerve (right). (**B1** to **B6**) Images of the surgical implantation procedures of the twining electrode. (**C** and **D**) Illustrations of the twining electrode that conformally contacts the deforming vagus nerve. (**E** to **G**) ECGs of the anesthetized rabbit in a normal state (E), after epinephrine injection (F), and during electrical stimulation (G). Photo credit: Yingchao Zhang, Tsinghua University.

In vivo VNS experiments on a rabbit animal model were first carried out to demonstrate the practical biomedical implications of the twining electrodes while simultaneously performing electrocardiography (ECG) (Fig. 5A). In addition to partial epilepsy and depression, VNS has also been proven to be effective in the treatment of chronic HF by improving the left ventricular function (*48*–*50*). We chose the twining electrode with an inner diameter of 1 mm, which equals to the diameter of the vagus nerve of the rabbit (Fig. 5A). First, the twining electrode was temporarily flattened to a 2D planar state for the convenience of handling before the surgical implantation. After exposing the vagus nerve (see Materials and Methods for details), we implanted the temporarily flattened electrode into the body to contact with the right vagus nerve, followed by the twining process driven by 37°C NS. The series of images in Fig. 5 (B1 to B6) and movie S6 display the detailed self-climbing process, which is similar to the climbing process of the flexible twining plants. In addition, the self-adaptive adjustment of the twining electrode was recorded, as shown in movie S7. In this way, the twining electrode can conformally contact with the vagus nerve and form good electrode-nerve interfaces even under extreme deformations (Fig. 5, C and D, and movie S8) without any additional fixation. Then, the normal ECG of the anesthetized rabbit was recorded after the implantation of the twining electrodes (Fig. 5E). The normal heart rate (HR) of the anesthetized rabbit was approximately 180 beats per minute (bpm). An animal model of HR variability was prepared by injection of epinephrine, leading to a change in HR that imitated the autonomic dysfunction. After the HR was stabilized, it increased from 180 to 240 bpm (Fig. 5F), indicating the excessive sympathetic activation. Next, according to previous studies (*9*, *49*, *51*), electrical stimulation with a constant current amplitude of 0.4 mA, a wave width of 100 μs, and a frequency of 10 Hz was applied to the right vagus nerve by the twining electrode. This VNS electrical neuromodulation, i.e., increasing the activation of the parasympathetic and concomitant withdrawal of the sympathetic, led to a decrease in HR from 240 bpm to the normal value of 180 bpm and an increase in the R-wave peak from 100 to 250 mV (Fig. 5G). Many studies in animal models and clinical patients have shown that the decrease in HR plays a notable role in the treatment of HF (*18*, *52*).

### Sciatic nerve action potential recording

**Fig. 6:**
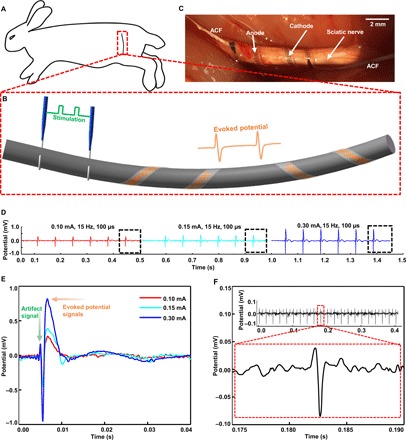
In vivo recording of the rabbit’s sciatic nerve using the twining electrodes. (**A** and **B**) Schematic diagram of the in vivo experimental setup. (**C**) Bipolar twining electrodes integrated on the sciatic nerve for recording. (**D**) Recorded CNAPs evoked by varying current (0.10, 0.15, and 0.3 mA). (**E**) Enlarged view of the comparison between the three evoked CNAPs. (**F**) Recorded CNAPs evoked by the shaking of the anesthetized rabbit’s leg (without electrical stimulation). Photo credit: Yingchao Zhang, Tsinghua University.

Next, we implanted the twining electrodes on the surface of the sciatic nerve of a rabbit and performed in vivo recording of action potential, which can be used as the feedback signals in the closed-loop control prosthetics and to study the relationship between the stimulation and response of the nerve (*2*, *31*, *53*). The experimental setup for stimulation and evoked potential recording is illustrated in Fig. 6 (A and B). The two hooked platinum electrodes (THPEs) were used for stimulation, and the twining electrodes were used for the recording. Figure 6C shows the twining electrodes that conformally contact the sciatic nerve in vivo, which were twined on the sciatic nerve by a process similar to that described previously. Then, a series of continuous monophasic rectangle waves of varying current (0.10 to 0.3 mA), a constant frequency of 15 Hz, and a wave width of 100 μs were delivered to the nerve through the THPEs. When the sciatic nerve was electrically stimulated, the evoked action potentials were transmitted along the nerve to the motor end-plates, enabling contraction of the relevant muscles and thus leading to the movement of the leg. The corresponding evoked compound nerve action potentials (CNAPs) that were recorded by the twining electrodes are shown in Fig. 6D, and one of the activated moments of the leg is shown in movie S9. All the recorded signals show stimulus artifact signals, followed by the evoked CNAPs. Both the amplitude and the waveform of the CNAPs are almost the same for each electrical stimulation. Figure 6E gives an enlarged view of the comparisons between the three evoked CNAPs. The waveforms are the same, while the peak potentials (i.e., ~275, ~380, and ~820 μV) increase with increasing applied current amplitudes (i.e., 0.1, 0.15, and 0.3 mA), which are consistent with the literature (*10*, *12*, *54*). In addition, after electrical stimulation, evoked CNAPs (peak potential, ~80 μV) without stimulus artifact signals corresponding to the shake of the anesthetized rabbit’s leg were also successfully recorded (Fig. 6F), showing a high SNR of 16 dB (note S4) due to the conformal contact with the nerve.

## DISCUSSION

The climbing-inspired concept introduced in this work forms a prototype, namely, the twining electrodes, which can self-climb onto the peripheral nerves by using the intelligent SMPs that respond to body temperature. The proposed twining electrodes can greatly reduce the nerve injury associated with the mechanical and geometrical mismatches and the surgical implantation, and thus show promising potential for electrical neural stimulation and recording in both clinical practice and basic neuroscience research. Furthermore, the processing paradigms proposed to match the 3D biological structures are compatible with the traditional 2D planar processing technology. Future twining electrodes will benefit from the introduction of new materials with high surface area and of high-resolution designs for selective stimulation and recording.

## MATERIALS AND METHODS

### Synthesis of the body temperature–driven SMPs

The polyurethane SMPs were synthesized by the reaction of three commercial precursors (fig. S2): polycaprolactone diol (PCL; *M*_w_ = 2000 g/mol; Sigma-Aldrich), poly(hexamethylene diisocyanate) (PHMD; *M*_w_ = 504 g/mol; Sigma-Aldrich), and hexamethylene diisocyanate (HDI; *M*_w_ = 168 g/mol; J&K Scientific Ltd.). Specifically, 2.0 g of PCL was weighted into a glass bottle and melted by heating in an oven at 100°C, before which PCL was dried with a vacuum freeze dyer for 24 hours. Then, 2 ml of butyl acetate (J&K Scientific Ltd.), PHMD, HDI (weight ratio, PHMD/HDI = 1:3), and 0.5 wt % (weight %) stannous octoate [Sn(Oct)_2_; *M*_w_ = 405.12 g/mol; Sigma-Aldrich] were added in sequence and stirred for several minutes. The mixture was poured into an aluminum mold (thickness, 100 μm) and cured at 60°C for 2 hours. Last, the cured sample film was vacuum-dried (80°C) overnight to obtain the initial planar SMP film.

### Thermomechanical and mechanical characterization of SMPs

Differential scanning calorimetry (Q200, TA Instruments) analyses were carried out at a cooling rate of 10°C/min. Dynamic mechanical analyses (DMAs; Q800, TA Instruments) were conducted under “multifrequency, strain” mode at 1 Hz, 0.2% strain, and a heating rate of 3°C/min. The quantitative consecutive elasticity (shape memory cycles) and the stress relaxation (plasticity or reconfigurability) experiments were carried out with the same DMA machine in a “force-controlled” and “displacement-controlled” mode, respectively. The “stress-strain” curve was obtained through the traditional tensile experiments (Zwick/Roell Z005).

### Film casting

Before the film casting, the silicon wafer was cleaned with acetone, ethyl alcohol, and deionized (DI) water. Then, the DI water was blown off with nitrogen, followed by heating and drying at 130°C for 5 min. A layer of PMMA (MicroChem) was spin-cast onto the silicon wafer at 900 rpm for 15 s and 300 rpm for 30 s and then cured at 120°C for 10 min, 150°C for 10 min, and 180°C for 20 min. After that, a layer of PI (Durimide; Fujifilm, China) was spin-cast at 900 rpm for 15 s and 4000 rpm for 40 s, and the PI film was cured at 80°C for 15 min, 100°C for 15 min, 120°C for 30 min, and 150°C for 70 min.

### Metal deposition and mesh serpentine design

Layers of 10-nm Ti and 200-nm Au were deposited onto the surface of the cured PI film using electron beam evaporation, and the deposition rate of the metal layer was controlled at ~0.12 nm/s. The Au/Ti layer was photolithographed and etched into the designed mesh serpentine patterns. Then, the PI film was patterned to the same shapes by RIE masked by Au (power, 200 W; pressure, 250 mtorr; O_2_, 30) for 1200 s.

### Electrochemical characterization

The electrochemical experiments of the electrodes were conducted on a CS350-type electrochemical workstation (CorrTest Instruments, China). A conventional three-electrode setup was used, where the twining electrode, Pt electrode, and silver/silver chloride (Ag/AgCl) were used as the working electrode, counter electrode, and reference electrode, respectively, in phosphate-buffered saline (pH 7.2 to 7.4) at room temperature (~20°C). EIS experiments were performed in a frequency range of 0.1 Hz to 100 kHz with a sinusoidal wave with amplitude of 10 mV. The CV experiments were performed from −0.6 to +0.8 V at a scan rate of 50 mV/s. Six samples were tested independently in four different states, i.e., initial flat state, reconfigured state, flattened state, and recovery state, which correspond to Fig. 2, D, F, G, and H, respectively. The inner radius is 0.5 mm. The CDC was defined as $\text{CDC}\;=\frac{1}{v}\int_{V_{a}}^{V_{c}}|i|dV$, where *v* is the scan rate; *V*_a_ and *V*_c_ are the anodic and cathodic potential limits (i.e., −0.6 to +0.8 V), respectively; *i* is the measured current density (mA/cm^2^); and *V* is the electrode potential versus Ag/AgCl.

### Finite element analyses

Finite element stimulations (Abaqus) were used to analyze the stress that was applied on the nerve by four different forms of extraneural electrodes, namely, traditional cuff electrode, traditional helical electrode, non-meshed twining electrode, and optimized twining electrode (fig. S9A), under three deformations (20% swelling, 20% stretching, and bending to *R* = 15 mm). The incompressible Neo-Hooke model was used to represent the substrate and the nerve with the parameter *C*_1_ = *E*_substrate,nerve_/6, linear elasticity was used to model the PI film, and the perfectly elastic-plastic model was used to model the Au layer. The detailed parameters used for FEA are summarized in fig. S9B. The extraneural electrodes were tied onto the nerve to simplify the FEA. For the bending and stretching deformations that were used to imitate the deformation of nerve during the movement, yield strain of Au (0.3%) was used for the design because the Au layer was subjected to cyclic loading. For the swelling deformation that was used to imitate the monotonous and slow growth process, fracture strain of Au (5%) was used for the design of the electrode because the swelling process was monotonous and the electrode was not subjected to cyclic deformation.

### In vivo animal experiments

All the animal experiments were performed in Beijing Medical Services Biotechnology (Beijing, China) and approved by the Ethics Committee of Beijing Medical Services Biotechnology (MDSW-2018-018C). Two New Zealand rabbits (3.2 to 3.3 kg) were anesthetized by intraperitoneal injection of xylazine hydrochloride (5 mg/kg), and anesthesia was maintained with isoflurane (2%)/oxygen for VNS and sciatic nerve action potential recording experiments, respectively. After being placed in the supine position with its head tilted toward the left, the rabbit was shaved near the right cervical region, followed by an ~10-cm skin incision and dissection through the subcutaneous tissues until the vagus nerve was visible. Then, the twining electrode was placed on the right vagus nerve, as described in the main text, to perform the VNS animal experiments. Similarly, the other rabbit was placed in the prone position and shaved near the buttock area, followed by an ~10-cm skin incision and dissection of the gluteus muscles to expose the sciatic nerve. The impedance of the electrode-nerve system was about ~1.9 kilohms. Then, the commercial THPEs (Xi’an Friendship Medical Electronics Co. Ltd.) and the twining electrodes were placed on the sciatic nerve for the evoked potential recording experiments. The reference (i.e., ground) electrode was placed near the skin incision. A biological data acquisition and stimulation system (BL-420F, Chengdu Techman Software) was used for nerve stimulation and recording as well as ECG recording.

## Supplementary Material

http://advances.sciencemag.org/cgi/content/full/5/4/eaaw1066/DC1

Download PDF

Movie S1

Movie S2

Movie S3

Movie S4

Movie S5

Movie S6

Movie S7

Movie S8

Movie S9

## SUPPLEMENTARY MATERIALS

Supplementary material for this article is available at http://advances.sciencemag.org/cgi/content/full/5/4/eaaw1066/DC1 Note S1. Recoverability of the twining electrode and the maximum strain in the Au layer. Note S2. Comparison of bending stiffness. Note S3. Comparison of tension stiffness. Note S4. Calculation of the SNR. Fig. S1. Design of the SMP network and the mechanistic illustration of reconfiguration (plastic) and recovery (elastic). Fig. S2. Chemical network of the precursor monomers and the synthesized SMPs. Fig. S3. Characterization of the thickness of the Au/Ti and PI layers. Fig. S4. Characterization of the SMP. Fig. S5. Cyclic voltammogram. Fig. S6. Impedance spectroscopy. Fig. S7. Mechanical model for the twining electrode and the corresponding results. Fig. S8. FEA models and results for *EA*. Fig. S9. The parameters used in the FEA and the corresponding FEA model. Fig. S10. The FEA comparison results of the normal and shear stress applied on the nerve under three deformation modes. Fig. S11. Calculations of the recorded SNR. Table S1. Comparison of (*EI*)_Twining_ and (*EI*)_Tradition_. Movie S1. Twining plants under complex deformations. Movie S2. The twining electrode is twined on a glass rod driven by 37°C water. Movie S3. The electrical conductivity test. Movie S4. The demonstration of the recovery of the twining electrode upon physiology temperature. Movie S5. The illustration of the mechanical reliability of the twining electrode under stretching and bending. Movie S6. The in vivo self-climbing on vagus nerve process of the twining electrode. Movie S7. The self-adaptive adjustment of the twining electrode. Movie S8. The twining electrode conformally contacts with the deforming vagus nerve. Movie S9. The activated moments of the leg of the anesthetized rabbit.
