# Supplementary material for: Climbing-inspired twining electrodes using shape memory for peripheral nerve stimulation and recording
Source: Sci Adv. 2019 Apr 19;5(4):eaaw1066. doi: 10.1126/sciadv.aaw1066 (PMC6505533; doi:10.1126/sciadv.aaw1066)
Supplement: http://advances.sciencemag.org/cgi/content/full/5/4/eaaw1066/DC1 [file supp_5_4_eaaw1066__index.html]

Science Advances | Science Advances

## Supplementary Materials

**The PDF file includes:**

- Note S1. Recoverability of the twining electrode and the maximum strain in the Au layer.
- Note S2. Comparison of bending stiffness.
- Note S3. Comparison of tension stiffness.
- Note S4. Calculation of the SNR.
- Fig. S1. Design of the SMP network and the mechanistic illustration of reconfiguration (plastic) and recovery (elastic).
- Fig. S2. Chemical network of the precursor monomers and the synthesized SMPs.
- Fig. S3. Characterization of the thickness of the Au/Ti and PI layers.
- Fig. S4. Characterization of the SMP.
- Fig. S5. Cyclic voltammogram.
- Fig. S6. Impedance spectroscopy.
- Fig. S7. Mechanical model for the twining electrode and the corresponding results.
- Fig. S8. FEA models and results for *EA*.
- Fig. S9. The parameters used in the FEA and the corresponding FEA model.
- Fig. S10. The FEA comparison results of the normal and shear stress applied on the nerve under three deformation modes.
- Fig. S11. Calculations of the recorded SNR.
- Table S1. Comparison of (*EI*)Twining and (*EI*)Tradition.

Download PDF

**Other Supplementary Material for this manuscript includes the following:**

- Movie S1 (.mp4 format). Twining plants under complex deformations.
- Movie S2 (.mp4 format). The twining electrode is twined on a glass rod driven by 37°C water.
- Movie S3 (.mp4 format). The electrical conductivity test.
- Movie S4 (.mp4 format). The demonstration of the recovery of the twining electrode upon physiology temperature.
- Movie S5 (.mp4 format). The illustration of the mechanical reliability of the twining electrode under stretching and bending.
- Movie S6 (.mp4 format). The in vivo self-climbing on vagus nerve process of the twining electrode.
- Movie S7 (.mp4 format). The self-adaptive adjustment of the twining electrode.
- Movie S8 (.mp4 format). The twining electrode conformally contacts with the deforming vagus nerve.
- Movie S9 (.mp4 format). The activated moments of the leg of the anesthetized rabbit.

**Files in this Data Supplement:**

- Adobe PDF - aaw1066\_SM.pdf
