# Supplementary material for: Climbing-inspired twining electrodes using shape memory for peripheral nerve stimulation and recording
Source: Sci Adv. 2019 Apr 19;5(4):eaaw1066. doi: 10.1126/sciadv.aaw1066 (PMC6505533; doi:10.1126/sciadv.aaw1066)
Supplement: Download PDF [file aaw1066_SM.pdf]

Science Advances
AAAS

advances.sciencemag.org/cgi/content/full/5/4/eaaw1066/DC1

Supplementary Materials for

Climbing-inspired twining electrodes using shape memory for peripheral nerve stimulation and recording

Yingchao Zhang, Ning Zheng, Yu Cao, Fengle Wang, Peng Wang, Yinji Ma, Bingwei Lu, Guohui Hou, Zizheng Fang, Ziwei Liang, Mengkun Yue, Yan Li, Ying Chen, Ji Fu, Jian Wu, Tao Xie, Xue Feng*

*Corresponding author. Email: fengxue@tsinghua.edu.cn

Published 19 April 2019, *Sci. Adv.* **5**, eaaw1066 (2019)
DOI: 10.1126/sciadv.aaw1066

The PDF file includes:

Note S1. Recoverability of the twining electrode and the maximum strain in the Au layer.
Note S2. Comparison of bending stiffness.
Note S3. Comparison of tension stiffness.
Note S4. Calculation of the SNR.
Fig. S1. Design of the SMP network and the mechanistic illustration of reconfiguration (plastic) and recovery (elastic).
Fig. S2. Chemical network of the precursor monomers and the synthesized SMPs.
Fig. S3. Characterization of the thickness of the Au/Ti and PI layers.
Fig. S4. Characterization of the SMP.
Fig. S5. Cyclic voltammogram.
Fig. S6. Impedance spectroscopy.
Fig. S7. Mechanical model for the twining electrode and the corresponding results.
Fig. S8. FEA models and results for *EA*.
Fig. S9. The parameters used in the FEA and the corresponding FEA model.
Fig. S10. The FEA comparison results of the normal and shear stress applied on the nerve under three deformation modes.
Fig. S11. Calculations of the recorded SNR.
Table S1. Comparison of $(EI)_{Twining}$ and $(EI)_{Tradition}$.

Other Supplementary Material for this manuscript includes the following:

(available at advances.sciencemag.org/cgi/content/full/5/4/eaaw1066/DC1)

Movie S1 (.mp4 format). Twining plants under complex deformations.
Movie S2 (.mp4 format). The twining electrode is twined on a glass rod driven by 37°C water.

Movie S3 (.mp4 format). The electrical conductivity test.
Movie S4 (.mp4 format). The demonstration of the recovery of the twining electrode upon physiology temperature.
Movie S5 (.mp4 format). The illustration of the mechanical reliability of the twining electrode under stretching and bending.
Movie S6 (.mp4 format). The in vivo self-climbing on vagus nerve process of the twining electrode.
Movie S7 (.mp4 format). The self-adaptive adjustment of the twining electrode.
Movie S8 (.mp4 format). The twining electrode conformally contacts with the deforming vagus nerve.
Movie S9 (.mp4 format). The activated moments of the leg of the anesthetized rabbit.

Note S1. Recoverability of the twining electrode and the maximum strain in the Au layer.

During the high-temperature reconfiguration process (Fig. 2e,f), the SMP substrate and the PI are reconfigured to a helical shape with a radius of r_0 due to the thermo-plastic, while the Au remains in linear elastic. After the reconfiguration process, the twining electrode is flattened to facilitate the subsequent surgical implantation (Fig. 2g), the radius of the recovered twining electrode becomes r (the inset in Fig. 4a). The recoverability is defined as the radius ratio between the initial designed radius and the recoverable radius r_0/r , which can be derived through the principle of energy minimization. Figure S7a gives the mechanics model. To simplify the quantitative analysis, the cross-sectional geometry of the twining electrode is modeled as a three-layer structure, namely, the SMP substrate, *n* PI and *n* gold bricks. The stress distribution of the three layers can be given as

$$\sigma = \begin{cases} E_{Au}\dfrac{y}{\rho}, & y_0 - h_{Au} < y < y_0 \\ E_{PI}\dfrac{y}{\rho}, & y_0 - h_{Au} - h_{PI} < y < y_0 - h_{Au} \\ E_{SMP}\dfrac{y}{\rho}, & y_0 - h_{Au} - h_{PI} - h_{\mathrm{SMP}} < y < y_0 - h_{Au} - h_{PI} \end{cases} \tag{S1}$$

where E and h denote the Young's modulus and the thickness, and the subscript *Au*, *PI* and *SMP* are used to denote the Au, PI and the SMP substrate layers, respectively. y is the local coordinate that equals zero at the neutral axis , y_0 denotes the distance between the neutral axis and the top layer of Au, and ρ is denotes the curvature radius of the bending. The axial equilibrium gives

$$n\int_0^{h_{Au}} E_{Au}\frac{y}{\rho}bdy + n\int_{h_{Au}}^{h_{PI}} E_{PI}\frac{y}{\rho}bdy + \int_{h_{PI}}^{h_{SMP}} E_{SMP}\frac{y}{\rho}Bdy = 0 \tag{S2}$$

where b and B denote the width of the Au and the substrate, respectively.

Therefore, the distance between the neutral axis and the top Au layer can be derived as

$$y_0 = \frac{nbE_{Au}h_{Au}^2 + nbE_{PI}\left(2h_{Au}h_{PI} + h_{PI}^2\right) + BE_{SMP}\left[2\left(h_{Au} + h_{PI}\right)h_{SMP} + h_{SMP}^2\right]}{\left(2nbE_{Au}h_{Au} + 2nbE_{PI}h_{PI} + 2BE_{SMP}h_{SMP}\right)} \tag{S3}$$

The deformation energy of Au, PI and the SMP substrate during the recovery from the flattened state can be given as

$$U_{Au} = \frac{1}{2}E_{Au}nb\left[\frac{h_{Au}^3}{12} + \left(y_0 - \frac{h_{Au}}{2}\right)^2 h_{Au}\right]\left(\frac{1}{r}\right)^2 \tag{S4}$$

$$U_{PI} = \frac{1}{2}E_{PI}nb\left[\frac{h_{PI}^3}{12} + \left(y_0 - h_{Au} - \frac{h_{PI}}{2}\right)^2 h_{PI}\right]\left(\frac{1}{r_0} - \frac{1}{r}\right)^2 \tag{S5}$$

$$U_{SMP} = \frac{1}{2}E_{SMP}B\left[\frac{h_{SMP}^3}{12} + \left(y_0 - h_{Au} - h_{PI} - \frac{h_{SMP}}{2}\right)^2 h_{SMP}\right]\left(\frac{1}{r_0} - \frac{1}{r}\right)^2 \tag{S6}$$

where, U_{Au} , U_{PI} and U_{SMP} are denoted as the deformation energy of Au, PI and the SMP substrate, respectively. Therefore, the total energy can be given as

$$U_{total} = U_{Au} + U_{PI} + U_{SMP} \tag{S7}$$

Assuming that there is no energy loss of the SMP during the recovery process, the recoverable radius r can be obtained by minimizing the total energy with respect to r (i.e., $\partial U_{total}/\partial r = 0$) . Thus the recoverability of the twining electrode can be given as

$$\frac{r_0}{r}=\frac{E_{PI}nb\left[\frac{h_{PI}^3}{12}+\left(y_0-h_{Au}-\frac{h_{PI}}{2}\right)^2h_{PI}\right]+E_{SMP}B\left[\frac{h_{SMP}^3}{12}+\left(y_0-h_{Au}-h_{PI}-\frac{h_{SMP}}{2}\right)^2h_{SMP}\right]}{E_{PI}nb\left[\frac{h_{PI}^3}{12}+\left(y_0-h_{Au}-\frac{h_{PI}}{2}\right)^2h_{PI}\right]+E_{SMP}B\left[\frac{h_{SMP}^3}{12}+\left(y_0-h_{Au}-h_{PI}-\frac{h_{SMP}}{2}\right)^2h_{SMP}\right]+E_{Au}nb\left[\frac{h_{Au}^3}{12}+\left(y_0-\frac{h_{Au}}{2}\right)^2h_{Au}\right]} \quad \text{(S8)}$$

The maximum strain in the Au layer during the shape reconfiguration and memory recovery process is

$$\varepsilon_{Au}^{\max}=\frac{y_0}{r_0+y_0} \quad \text{(S9)}$$

Substituting the parameters of the optimized twining electrode, i.e., $E_{Au}=78\text{GPa}$, $E_{PI}=2.5\text{GPa}$, $E_{SMP}=300\text{kPa}$, $h_{Au}=200\text{nm}$, $nb=90\mu\text{m}$, $B=1\text{mm}$ into equations S8 and S9 gives the Fig. 4a,b and fig. S7b,c.

It should be noted that, there are always some differences between the diameters of the pre-designed twining electrodes and the peripheral nerves even we have known the approximate diameters of nerves:

(1) The first case is that the diameter of the electrode r_0 is a little smaller than the diameter of the nerve r_{nerve} (i.e., $r_0 < r_{\text{nerve}}$). A little compression will be applied on the nerve after the electrode was twined on, since our electrode has the tendency to coil to its initial shape (i.e., the helix with radius r_0) during the shape recovery process. This shape recovery ability can be very helpful for the good contact at the electrode-nerve interface. And most importantly, this compression that our electrode applied on the nerve is much more less than that of the traditional cuff electrode. Because the bending stiffness of our electrode (~1.0×10^{-10} N•m^2) is much more less than that of the traditional cuff electrode (~2.1×10^{-7} N•m^2) (See note S2).

(2) The second case is that r_0 is a little larger than r_{nerve} (i.e., $r_0 > r_{\text{nerve}}$). In this case, our twining electrode also has the tendency to recover to its initial shape, i.e., it does have a tendency to

separate from the electrode-nerve interface after it was twined on the nerve. However, it will keep good contact only if $U < \gamma$, where U is the deformation energy and γ is the interface adhesion energy. For the qualitative analysis, we can get that $U \approx \frac{1}{2} EI \left(\frac{1}{r_0} - \frac{1}{r_{\text{nerve}}} \right)^2$ and EI is the bending stiffness of the electrode. Therefore, if we want to increase the reliability of the electrode-nerve interface (i.e., keep good contact to avoid the delamination of the electrode from the nerve surface), we need to reduce the bending stiffness EI and diminish the difference between r_0 and r_{nerve}. The bending stiffness have been reduced from ~2.1×10^{-7} N•m^2 (traditional cuff electrode) and ~4.6×10^{-10} N•m^2 (PI-based extraneural electrodes) to ~1.0×10^{-10} N•m^2 (our electrode) (See note S2) by the structural optimization. Although the bending stiffness EI of the PI-based extraneural electrode is compatible with that of our twining electrode, the PI-based extraneural electrode is 2D, which means that the diameter is infinite and much more interfacial adhesion is required. This is why it often needs additional fixation, such as surgical suture (*9*). As for our twining electrode, whose permanent shape is 3D, less interfacial adhesion is required due to the minimized mechanical mismatch (i.e., *EI is smaller*) and geometrical mismatch (r_0 is only a little bigger than r_{nerve}).

Note S2. Comparison of bending stiffness.

The bending stiffness of the twining electrode can be given as

$$(EI)_{Twining} = nbh_{Au}E_{Au}\left[\frac{h_{Au}^2}{12} + \left(y_0 - \frac{h_{Au}}{2}\right)^2\right] + nbh_{PI}E_{PI}\left[\frac{h_{PI}^2}{12} + \left(y_0 - h_{Au} - \frac{h_{PI}}{2}\right)^2\right] + Bh_{SMP}E_{SMP}\left[\frac{h_{SMP}^2}{12} + \left(y_0 - h_{Au} - h_{PI} - \frac{h_{SMP}}{2}\right)^2\right] \quad \text{(S10)}$$

where, r_0 denotes the initial radius (i.e., the designed radius) of the twining electrode.

The EI of the traditional silicone rubber-based and the PI-based extraneural electrodes can be given as

$$\left(EI\right)_{Tradition}=nbh_{Metal}E_{Metal}\left[\frac{h_{Metal}^{2}}{12}+\left(y_0{'}-\frac{h_{Metal}}{2}\right)^{2}\right]+Bh_{Sub}E_{Sub}\left[\frac{h_{Sub}^{2}}{12}+\left(y_0{'}-h_{Metal}-\frac{h_{Sub}}{2}\right)^{2}\right] \quad \text{(S11)}$$

where

$$y_0{'}=\frac{nbE_{Metal}h_{Metal}^{2}+BE_{Sub}\left(2h_{Metal}h_{Sub}+h_{Sub}^{2}\right)}{\left(2nbE_{Metal}h_{Metal}+2BE_{Sub}h_{Sub}\right)} \quad \text{(S12)}$$

E and h are the modulus and the thickness of the traditional extraneural electrodes respectively, and the subscripts ‘Sub’ and ‘Metal’ denote the substrate layer and the metal layer, respectively. For the comparison between $(EI)_{\text{Twining}}$ and $(EI)_{\text{Tradition}}$, the related parameters are calculated as shown in the following table.

Table S1. Comparison of $(EI)_{\text{Twining}}$ and $(EI)_{\text{Tradition}}$.

	Thickness	Width	Modulus	*EI*
**Twining**	h_{Au}=200 nm, h_{PI}=2 μm, h_{SMP}=100 μm	nb=90 μm, B=1 mm	E_{Au}=78 GPa, E_{PI}=2.5 GPa, E_{SMP}=300 kPa	1.0×10^{-10} N•m^2
**Rubber-Based (*5,8,43*)**	h_{Pt}=25 μm, h_{sub}=100 μm	nb=1 mm, B=1 mm	E_{Pt}=160 GPa, E_{Sub}=2 MPa	2.1×10^{-7} N•m^2
**PI-Based (*9,43*)**	h_{Au}=200 nm, h_{PI}=10 μm	nb=1 mm, B=1 mm	E_{Au}=78 GPa, E_{PI}=2.5 GPa,	4.6×10^{-10} N•m^2

Note S3. Comparison of tension stiffness.

Substitution of the same parameters mentioned above into the following equation

$$(EA)_{Tradition} = E_{Metal} h_{metal} nb + E_{Sub} h_{Sub} B \tag{S13}$$

gives that the *EA* of the traditional silicone rubber-based and the PI-based extraneural electrodes are 5000 N and 40 N respectively.

The *EA* of the twining electrode $(EA)_{\text{Twining}}$ is too complex to give an analytical expression. Therefore, we obtain $(EA)_{\text{Twining}}$ by adopting the FEA. Figure S8 illustrates the FEA models. Here, we analyze three different models to validate the FEA results. The FEA results show that the *EA* of the non-meshed, mesh and mesh-serpentine structures are 13.0 N, 1.87 N and 0.0807 N, respectively. The analytical solutions for the non-meshed and mesh structure are 13.008 N and 1.884 N respectively. This result also shows that, the mesh-serpentine design can markedly diminish *EA* by transforming the deformation mode from stretching-dominated to bending-dominated due to the initial curvature of the serpentine.

Note S4. Calculation of the SNR.

The recordings of the potentials evoked by the shaking of the anaesthetized rabbit's leg are divided into three parts (fig. S11 a), i.e., the noise-1, the signal, and the noise-2. Figure S11b gives the spectrograms of the three parts, which shows a high SNR. The SNR is defined as the ratio of the signal power to the noise power

$$SNR = 10\log_{10}\left(\frac{Signal\ power}{Noise\ power}\right) \tag{S14}$$

where

$$Signal\ power = \frac{1}{N_{Signal}}\sum_{n=0}^{N_{Signal}}\left|X_{Signal}\left(f\right)\right|^2 \tag{S15}$$

$$Noise\ power = \frac{1}{2}\left(\frac{1}{N_{Noise\text{-}1}}\sum_{n=0}^{N_{Noise\text{-}1}}\left|X_{Noise\text{-}1}\left(f\right)\right|^2 + \frac{1}{N_{Noise\text{-}2}}\sum_{n=0}^{N_{Noise\text{-}2}}\left|X_{Noise\text{-}1}\left(f\right)\right|^2\right) \tag{S16}$$

Therefore, we can find that the SNR of the evoked potentials is 16 dB.

Supplementary Figures

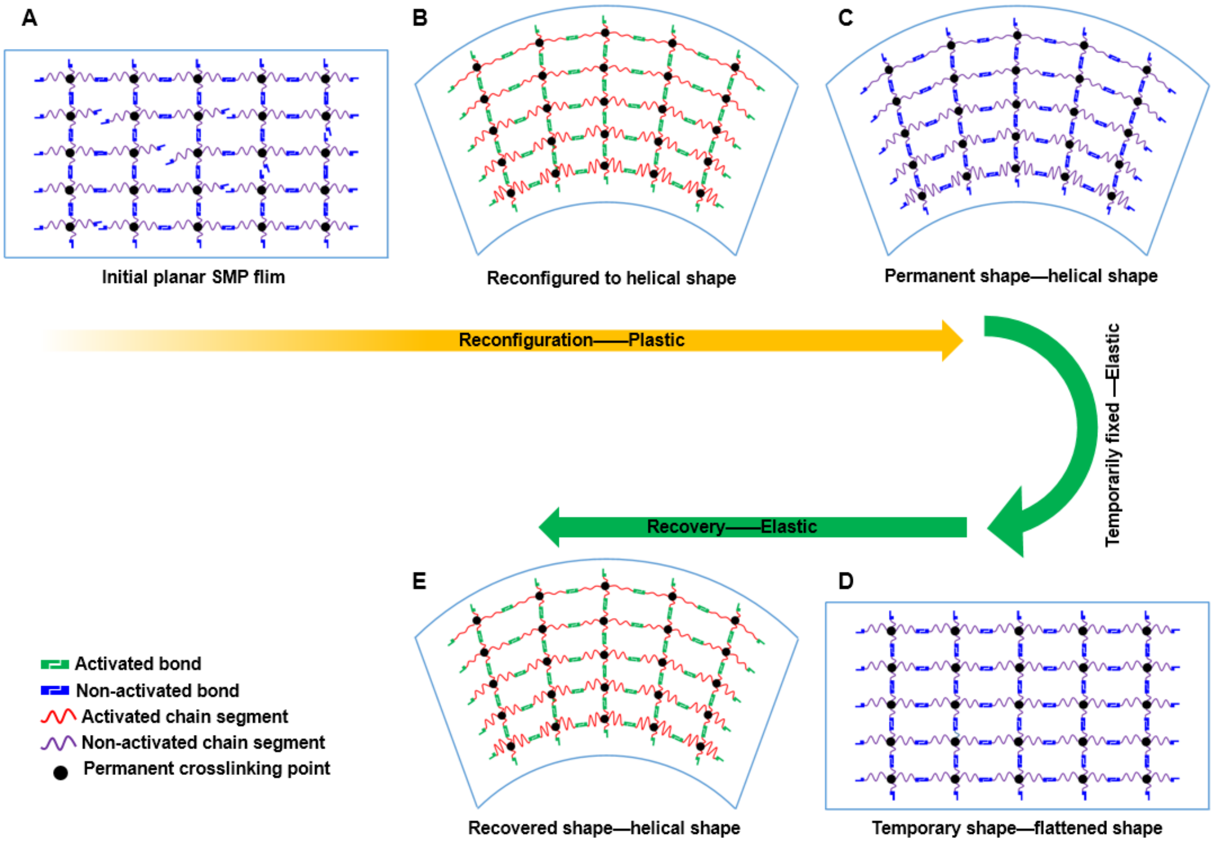

Fig. S1. Design of the SMP network and the mechanistic illustration of reconfiguration (plastic) and recovery (elastic). Figure S1(**A, B, C, D, E)** corresponds to the parts of the fabrication process shown in Fig. 2D, E, F, G, H in the main text.

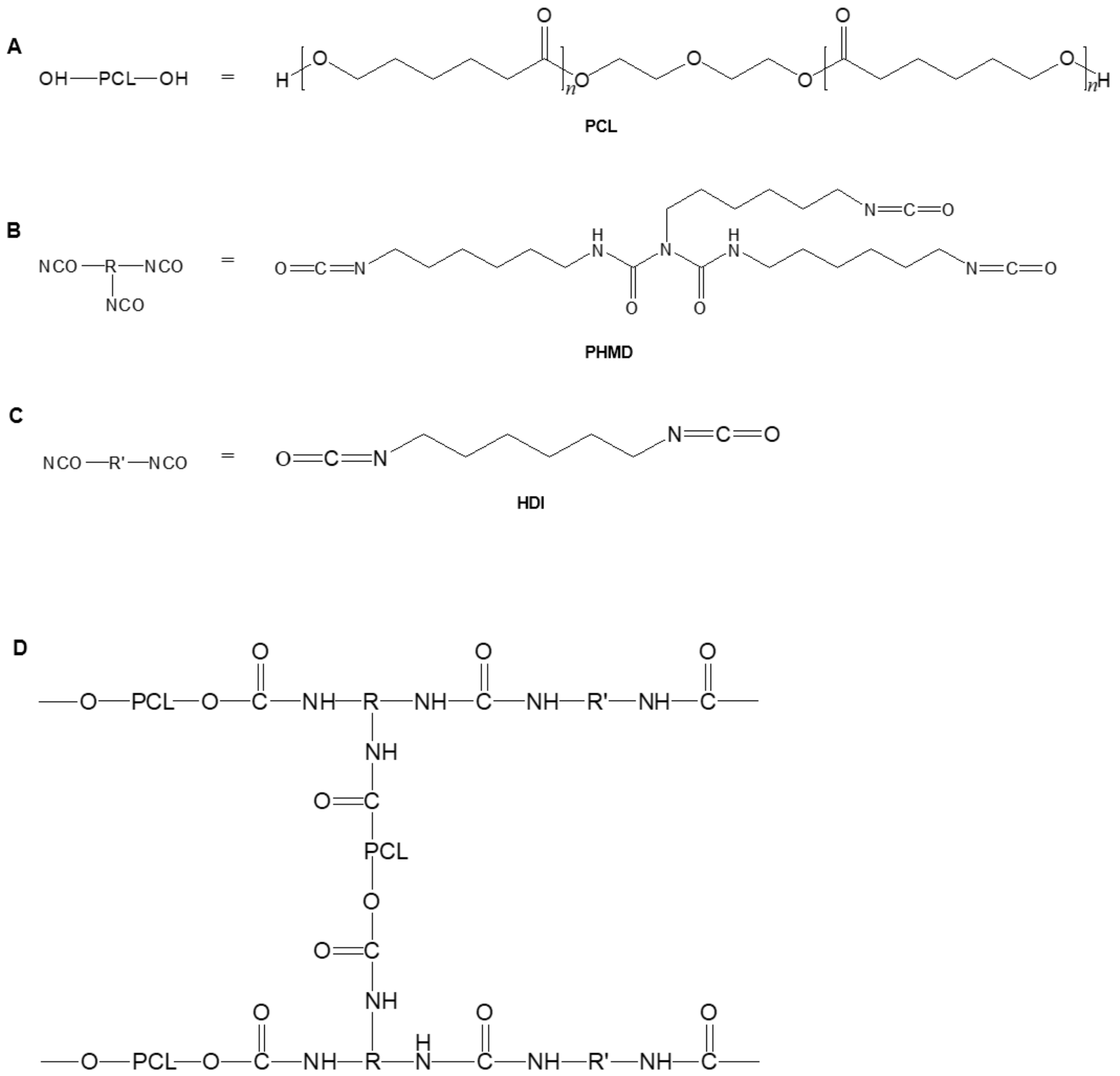

Fig. S2. Chemical network of the precursor monomers and the synthesized SMPs. (A) PCL. **(B)** PHMD. **(C)** HDI. **(D)** The synthesized SMPs.

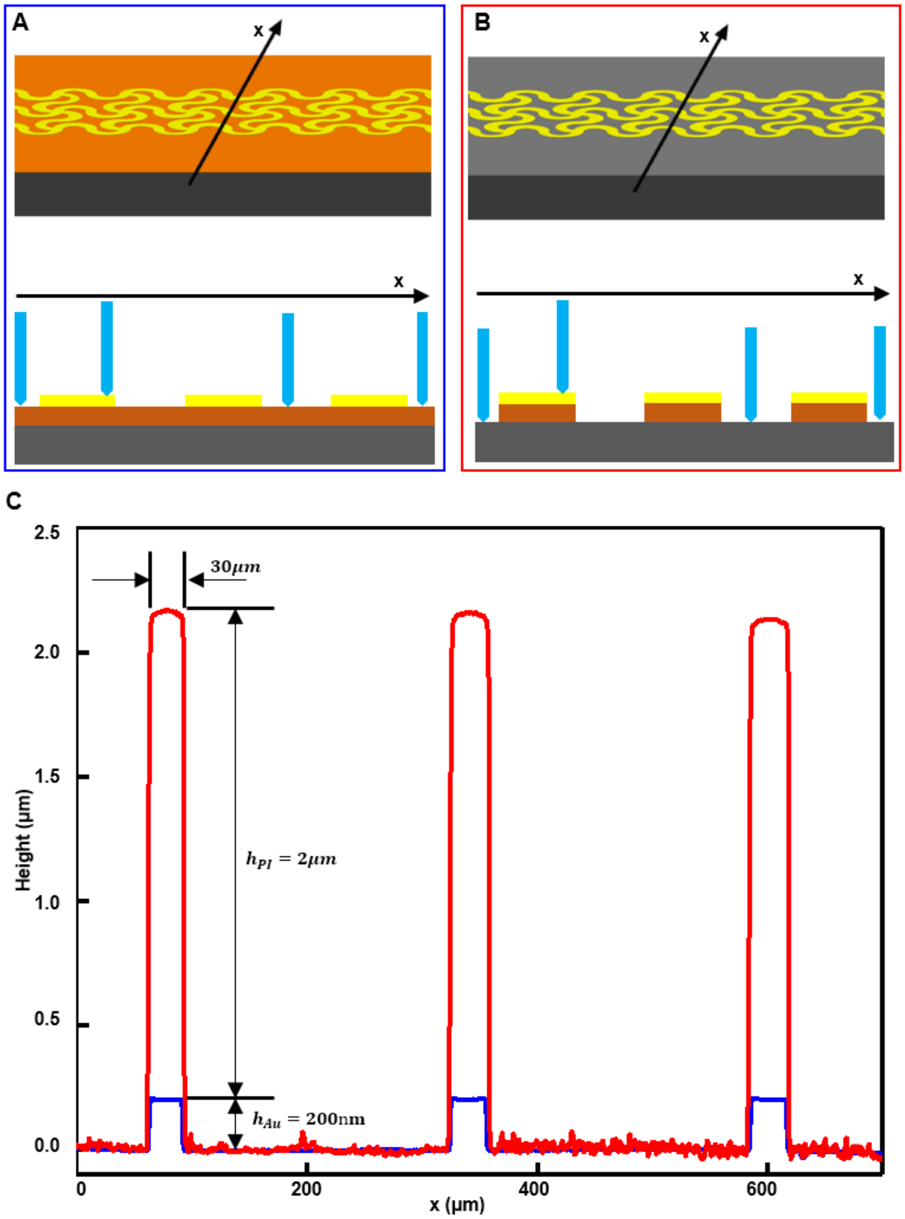

Fig. S3. Characterization of the thickness of the Au/Ti and PI layers. (A) and **(B)** Illustrations of the measurements by a profilometry system before and after RIE respectively. **(C)** The height measurements before and after RIE.

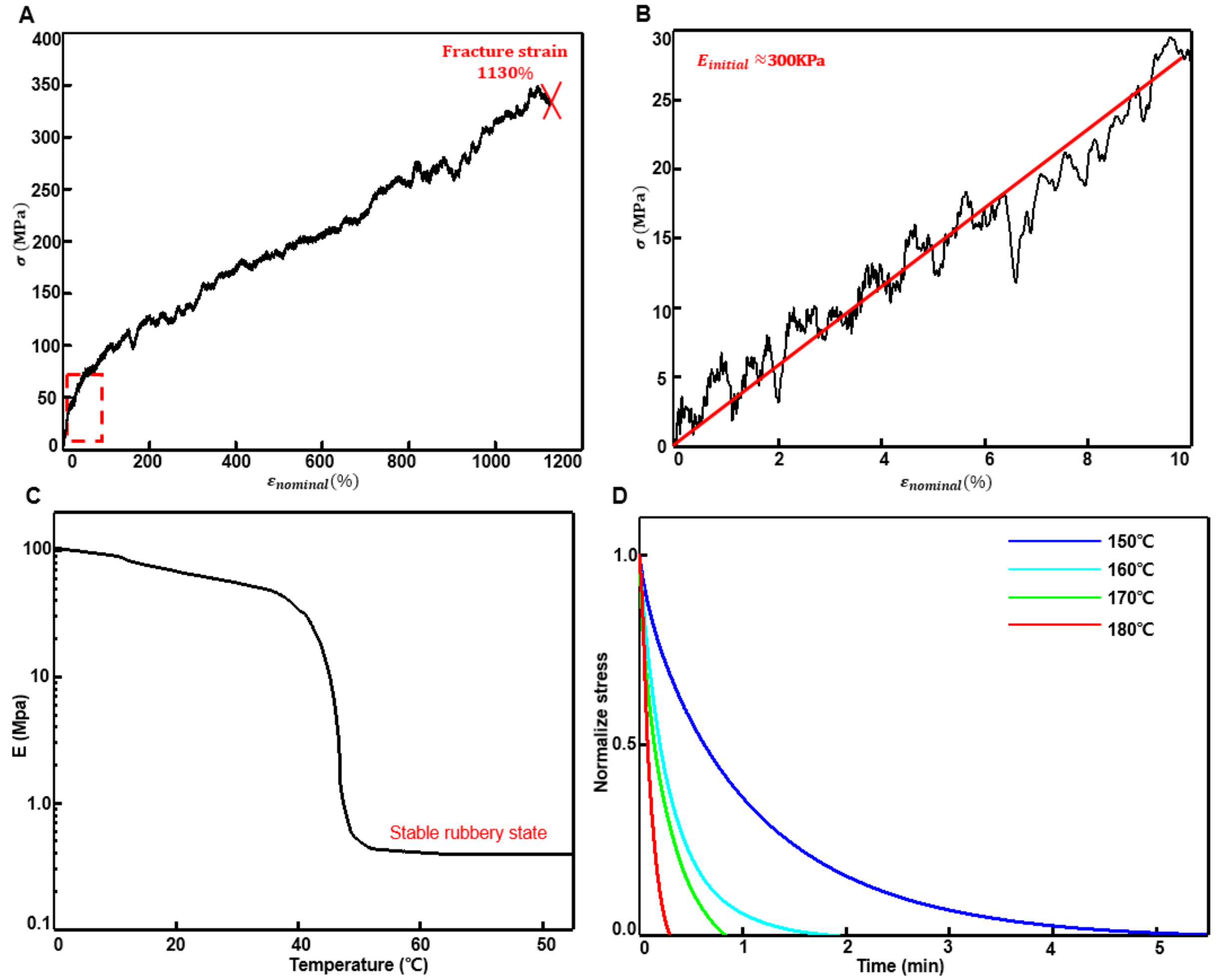

Fig. S4. Characterization of the SMP. (A) and **(B)** The stress-strain curve, which gives the fracture strain (~1100%) and the initial elastic modulus (~300 kPa). **(C)** The DMA curve. **(D)** The stress relaxation curves at different temperatures.

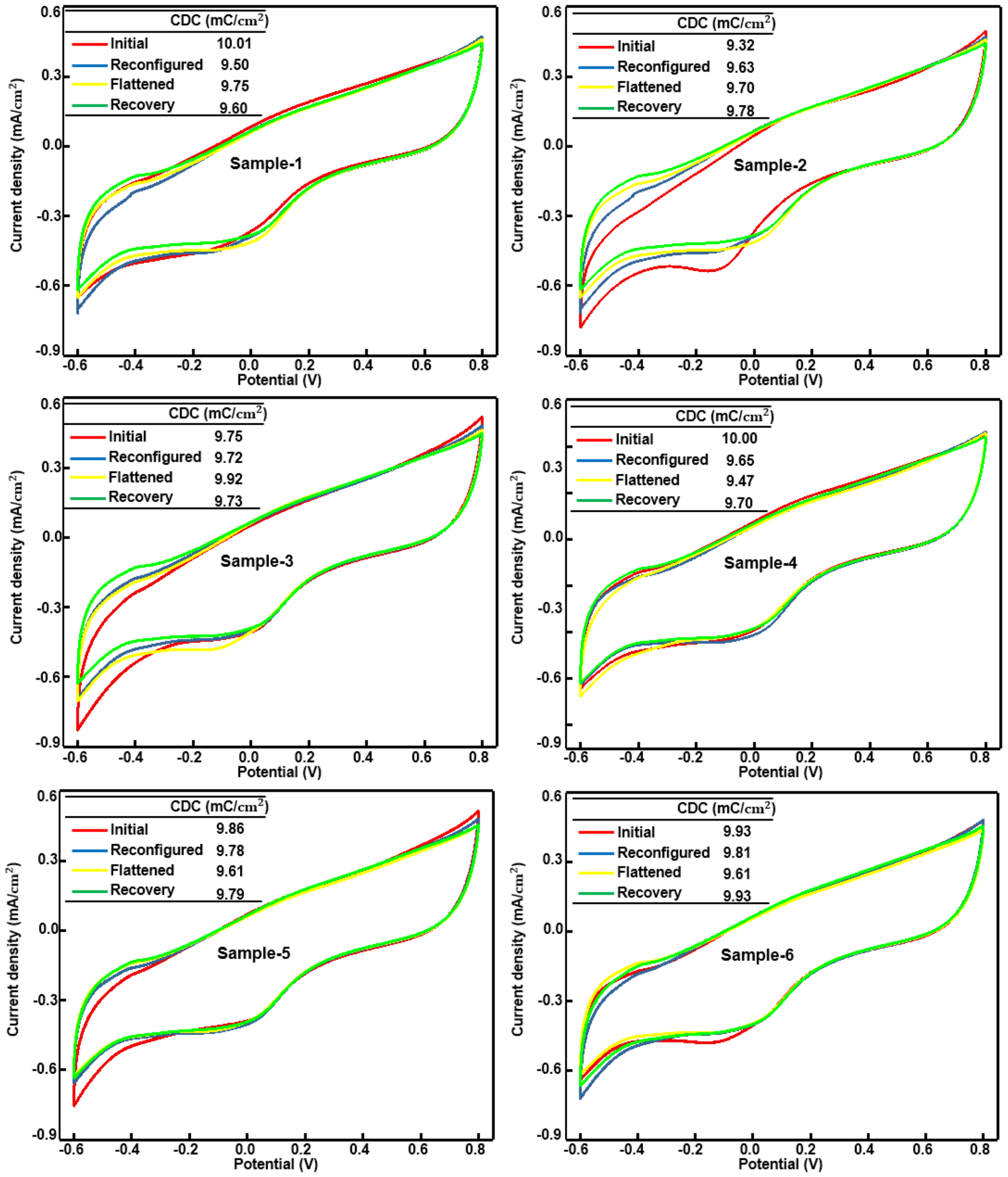

Fig. S5. Cyclic voltammogram. The CV curves of six samples under four different states, where the inset tables give the corresponding CDC values. The average CDC is ~9.7 mC/cm^2.

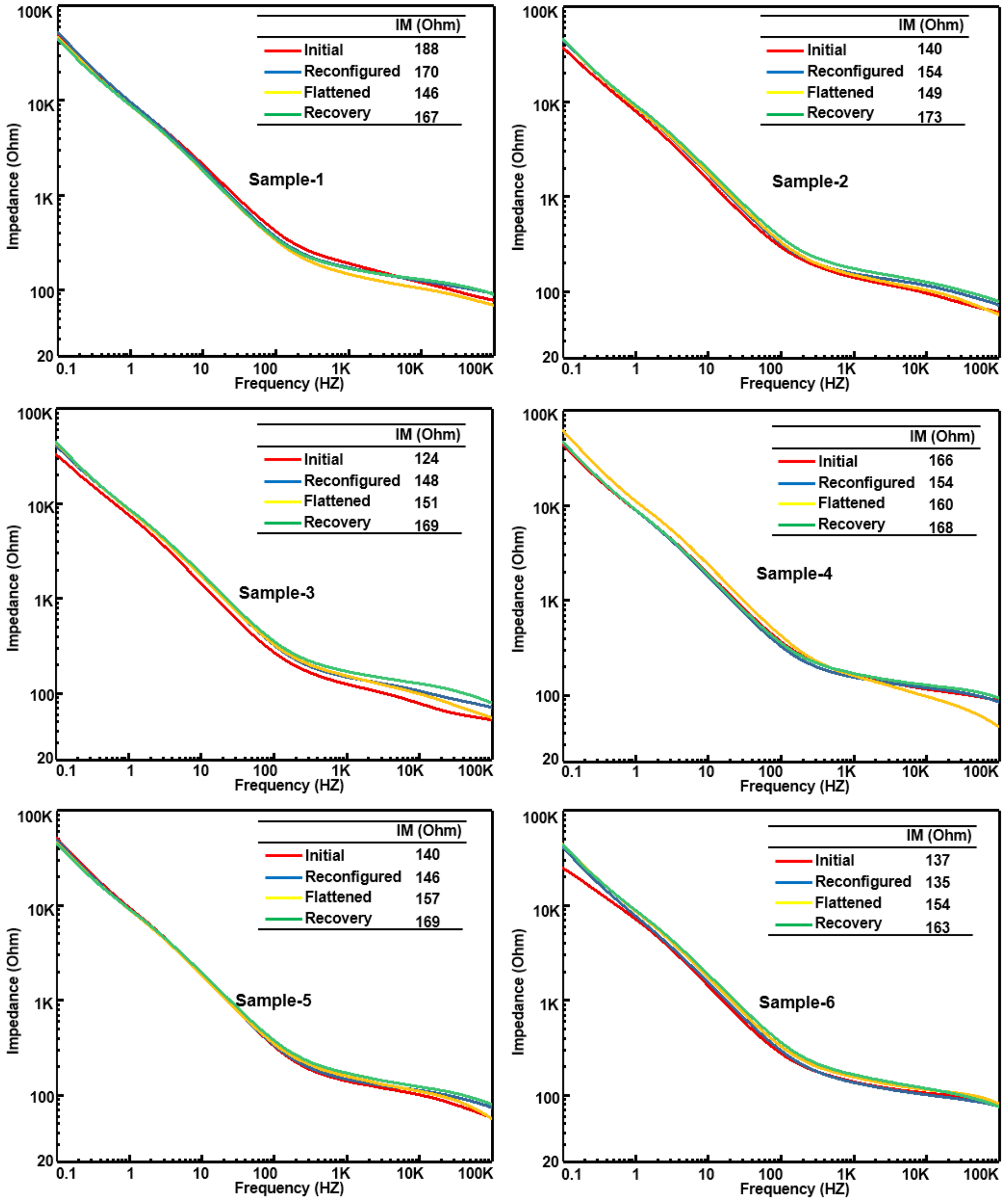

Fig. S6. Impedance spectroscopy. The impedance spectroscopy of six samples under four different states, where the inset figures gives the corresponding impedance magnitude values at 1 kHz. The average impedance magnitude at 1 kHz is 156 Ohm.

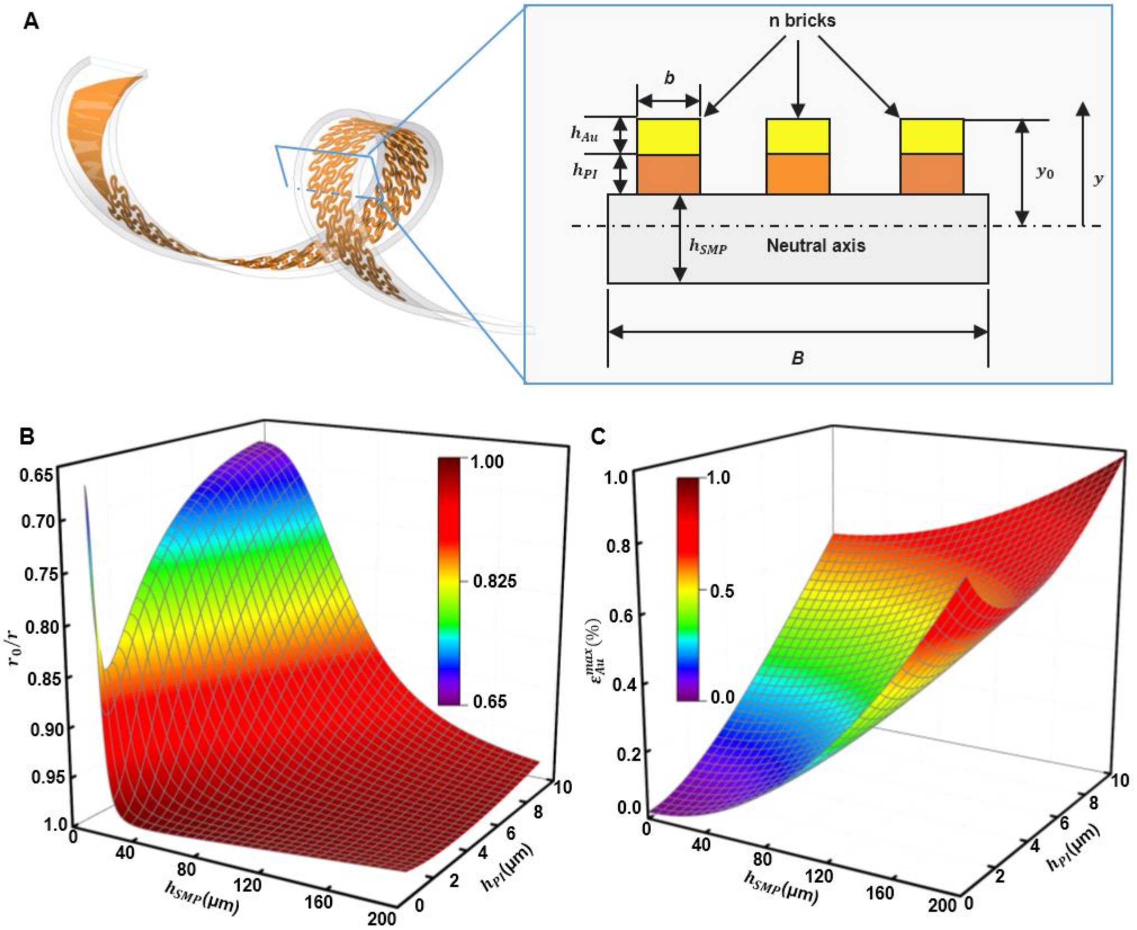

Fig. S7. Mechanical model for the twining electrode and the corresponding results. (A) The mechanics model. **(B)** and **(C)** r_0/r and ε_{Au}^{max} versus h_{SMP} and h_{PI}, respectively.

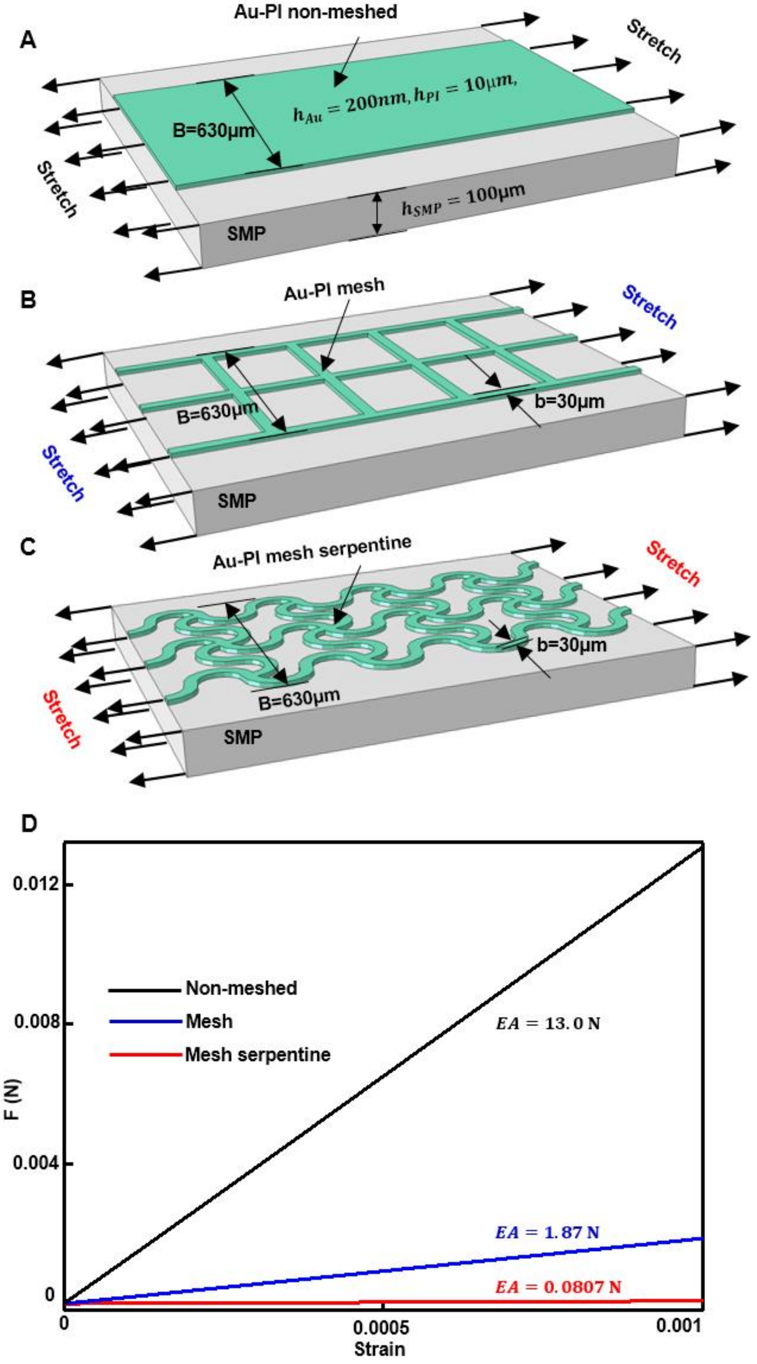

Fig. S8. FEA models and results for *EA*. (A), **(B)** and **(C)**, The FEA models of the Au-PI non-meshed design, the mesh design and the mesh-serpentine design respectively. **(D)** The tension F (N) versus the strain curves of the three designs.

A

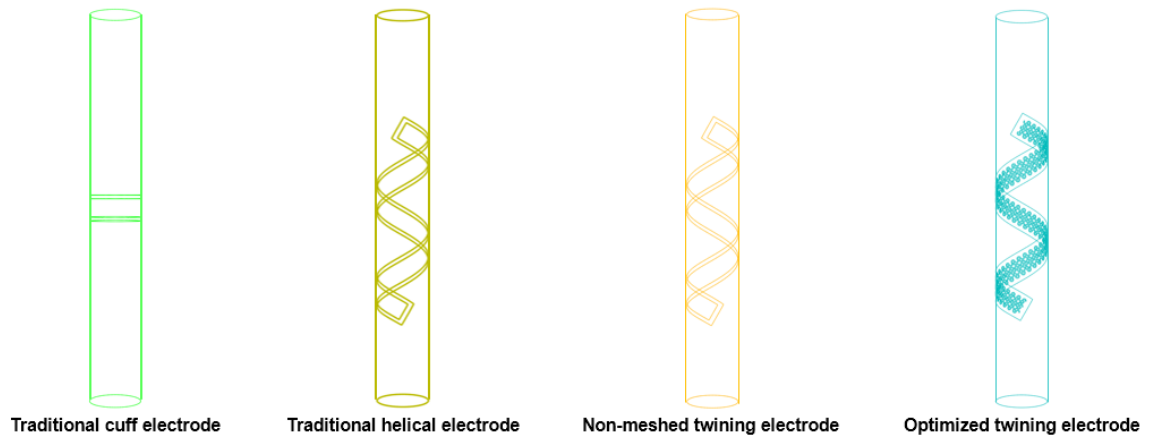

B

No.		Layer	Thickness	Property	Element type
1	Traditional cuff electrode	Substrate	100μm	E=2MPa, C1=0.33 (Neo Hooke)	S4R
		Au	25μm	E=78GPa, $\sigma_{yield} = 234\text{MPa}$ (Elastic-Plastic)	S4R
2	Traditional helical electrode	Substrate	200μm	E=2MPa, C1=0.33 (Neo Hooke)	S4R
		Au	25μm	E=2MPa, C1=0.33(Neo Hooke)	S4R
3	Non-meshed twining electrode	Substrate	100μm	E=300kPa, C1=0.05 (Neo Hooke)	S4R
		Au	200nm	E=78GPa, $\sigma_{yield} = 234\text{MPa}$ (Elastic-Plastic)	S4R
		PI	2μm	E=2.5GPa (Elastic)	S4R
4	Optimized twining electrode	Substrate	100μm	E=300kPa, C1=0.05 (Neo Hooke)	S4R
		Au	200nm	E=78GPa, $\sigma_{yield} = 234\text{MPa}$ (Elastic-Plastic)	S4R
		PI	2μm	E=2.5GPa (Elastic)	S4R
	Nerve			E=20kPa, C1=0.0033 (Neo Hooke)	C3D8RH

Fig. S9. The parameters used in the FEA and the corresponding FEA model. (A) the FEA models of the traditional cuff electrode, helical electrode, none-meshed twining electrode and optimized twining electrode. (**B)** Corresponding parameters.

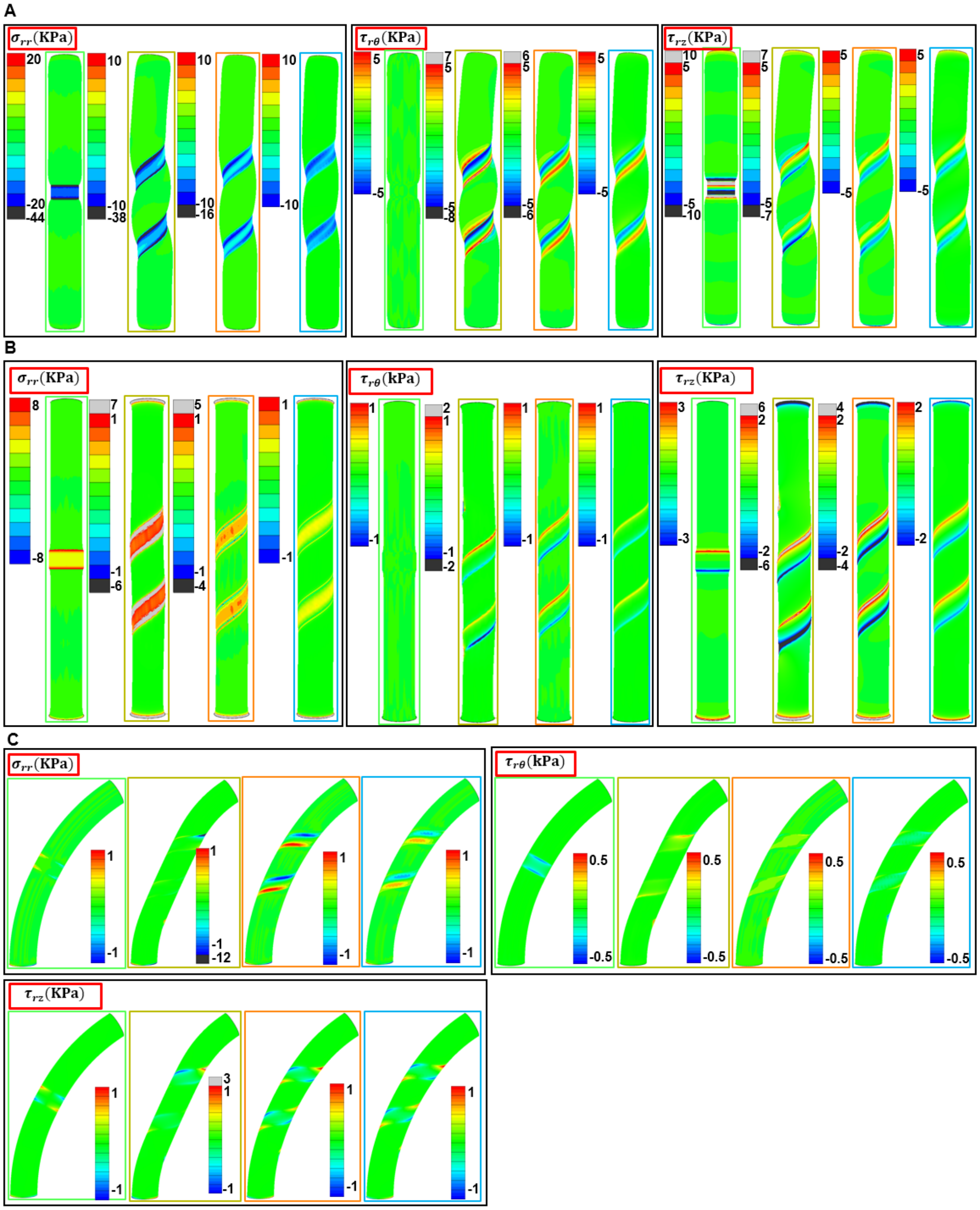

Fig. S10. The FEA comparison results of the normal and shear stress applied on the nerve under three deformation modes. (A) Swelling, 20%. **(B)** Stretching, 20%. **(C)** Bending, with a bending radius of 15 mm.

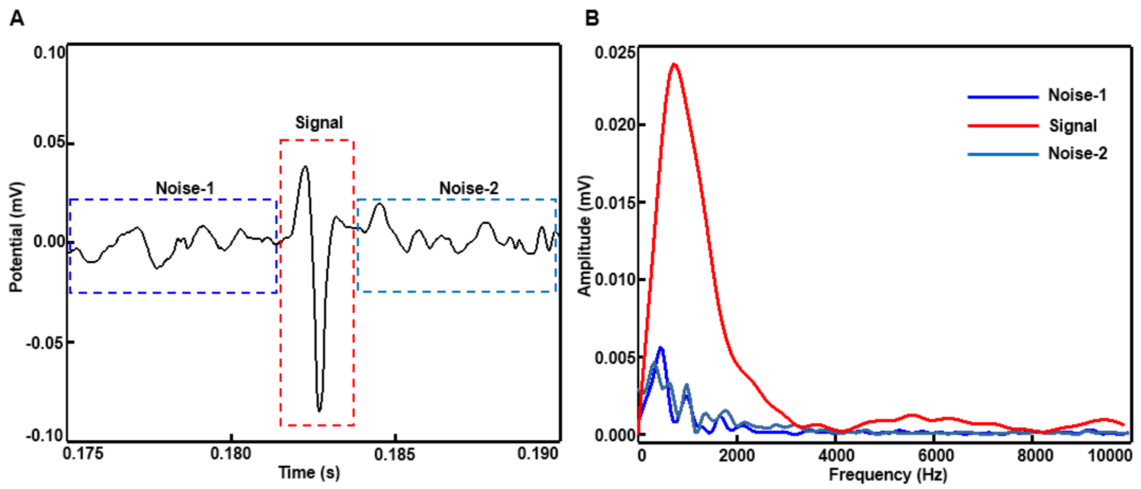

Fig. S11. Calculations of the recorded SNR. (A) The recordings are divided into three parts, i.e., the noise-1, the signal and the noise-2. **(B)** The corresponding spectrograms of the three parts.
